# Microbial shifts in early life: the pediatric gut microbiome and its role in health and disease

**DOI:** 10.1080/19490976.2026.2681763

**Published:** 2026-06-02

**Authors:** Sonali Waghmode, Rajlakshmi Viswanathan, Vishal Koligudde, Pooja Umare, Mallika Lavania

**Affiliations:** a ICMR-National Institute of Virology, Pune, India; b Academy of Scientific and Innovative Research (AcSIR), Ghaziabad, India

**Keywords:** Gut microbiome, virome, neurodevelopmental disorders, imbalanced gut, therapeutic

## Abstract

This review explores the pivotal role of the pediatric gut microbiome in shaping early-life development and influencing susceptibility to disease, emphasizing its impact on immune, metabolic, and neurodevelopmental processes. The neonatal period represents a critical window for host–microbiome interactions, beginning at birth when intestinal barrier function is still developing and immune responses remain immature. During this formative stage, rapid microbial colonization and ecological succession are influenced by delivery mode, infant feeding practices, antibiotic exposure, and environmental factors. Beyond bacterial populations, the early-life gut virome composed of bacteriophages and eukaryotic viruses evolves dynamically and contributes to microbial community structure, gene exchange, and immune system maturation. Microbially derived signals and metabolites support the development of mucosal integrity, immune programming, and host microbe equilibrium, with long-term implications for systemic immune function. Breastfeeding fosters the establishment of microbial communities and metabolic profiles associated with immune tolerance, whereas formula feeding and early-life antibiotic use may disrupt normal microbiome development. Alterations in early microbial trajectories have been associated with heightened risk of pediatric conditions, including allergic diseases, obesity, inflammatory bowel disease, and neurodevelopmental disorders. The review further evaluates emerging microbiome-directed strategies, such as probiotics, prebiotics, and fecal microbiota transplantation, considering both their therapeutic promise and current challenges. Collectively, current evidence underscores the early-life gut microbiome as a central determinant of host development and a compelling target for disease prevention strategies.

## 1. The early-life gut microbiome as an integrative regulator of immune, metabolic, and neurodevelopmental pathways

The human gut microbiome, a diverse community of microorganisms including bacteria, viruses, fungi, and archaea, plays an essential role in the development and function of the immune, metabolic, and central nervous systems. The human gut microbiome constitutes a dynamic, multi-kingdom ecosystem that interacts closely with host immune, metabolic, and neurodevelopmental pathways. In early life, this ecosystem undergoes rapid assembly during critical developmental windows, during which microbial signals, intestinal barrier maturation, and environmental exposures converge to program long-term health trajectories. While the specific composition of these microbes varies among healthy individuals, their functional capabilities remain largely consistent.^1^ ^,^ ^2^ In pediatrics, the early-life gut microbiome is particularly critical as it significantly influences a child’s health trajectory. From birth through infancy and into early childhood, the gut microbiome undergoes profound changes, shaping both physiological and immunological development.^3^ As the gut microbiome matures, it helps in the establishment of a balanced immune system, protection against harmful pathogens, and proper regulation of metabolism. Conversely, disruptions in this process, commonly referred to as imbalanced gut, are associated with a wide range of diseases, including autoimmune conditions, allergies, obesity, and even neurodevelopmental disorders.^4^ ^,^ ^5^ The early-life microbiome is not static; rather, it is influenced by numerous factors, such as the mode of delivery, feeding practices, antibiotic exposure, and environmental interactions. At birth, the infant's gut is sterile, and its microbiome begins to develop immediately after delivery.^6^ The mode of birth significantly impacts the first microbial communities that colonize the infant’s gastrointestinal (GI) tract. Vaginal delivery introduces maternal vaginal and fecal microbes, whereas cesarean section leads to a different microbial colonization primarily derived from the hospital environment.^7^ A study shows that infant gut and oral microbiota composition in the first month of life is strongly shaped by delivery mode, with vaginal birth associated with taxa also present in parental microbiota. While microbial overlap between infants and parents was observed, limited taxonomic resolution precluded clear differentiation of maternal versus paternal contributions, highlighting the need for higher-resolution approaches.^8^ A study of Bangladeshi mother–infant dyads during early infancy revealed age-related increases in microbially derived bile and lipid metabolites, reflecting contributions from both maternal and environmental microbial seeding. Delivery mode, milk composition, and household conditions particularly cesarean section birth and untreated drinking water were associated with transient yet distinct infant gut metabolic signatures.^9^

Additionally, breastfeeding provides a rich source of beneficial bacteria and immune-modulating factors like human milk oligosaccharides (HMOs), which foster the growth of specific beneficial microbiota, such as *Bifidobacterium* species, while formula feeding typically leads to a different microbial profile.^10^ Moreover, the GI barrier in infants is more permeable than in adults, which allows for the translocation of larger molecules, including immunoglobulins, across the intestinal epithelium, offering passive immune protection but also raising the risk of infections.^11^ During this time, the gut microbiome plays a pivotal role in stimulating immune system development, particularly by helping to establish immune tolerance and promoting the maturation of mucosal immunity.^12^ Early nutrition is a critical determinant of microbiome composition and immune system development. Breastfeeding, for example, has been shown to influence the gut microbiome by promoting the growth of beneficial bacteria that contribute to the development of a balanced immune system,^13^ as well as lower rates of allergies, asthma, and autoimmune diseases.^14^

The early use of antibiotics can disrupt the developing microbiome, leading to a reduction in microbial diversity and an increased risk of conditions like obesity, allergies, and GI diseases later in life.^15^ ^,^ ^16^

As the infant gut microbiome matures, it interacts with the immune system to establish a healthy, well-functioning defense network. Gut microbiota helps educate the immune system by promoting the development of both innate and adaptive immune responses.^17^ Furthermore, they contribute to the establishment of the gut barrier, which prevents pathogens from entering the bloodstream and causing infection.^18^ Imbalanced gut, has been implicated in a range of pediatric diseases, including inflammatory bowel disease (IBD), food allergies, asthma, obesity, and neurodevelopmental disorders such as autism spectrum disorder (ASD).^19^ ^,^ ^20^ Studies have shown that children with IBD have a significantly altered microbiome, with a reduction in beneficial microbes like *Faecalibacterium prausnitzii* and an overgrowth of harmful bacteria such as *Escherichia coli*.^21^ Similarly, imbalanced gut has been linked to the development of allergies and asthma, with a reduction in diversity and a predominance of pro-inflammatory microbial species.^22^

In recent years, researchers have identified the gut-brain axis, a bidirectional communication pathway between the gut microbiome and the central nervous system, which plays a significant role in neurodevelopment. Studies have shown that children with autism spectrum disorder (ASD) often exhibit microbiome alterations, including reduced diversity and an overrepresentation of certain bacterial taxa.^23^ A study links ASD to distinct interactions between diet quality, synthetic food additives, and gut microbiome composition in children. Children with ASD showed heightened microbiome sensitivity to poor diets and emulsifier exposure, which correlated with core autistic symptoms and GI issues.^24^ These changes may contribute to both GI symptoms and the neurobehavioral manifestations of ASD, highlighting the far-reaching impact of the microbiome on both gut and brain health.^25^ Emerging research into the role of the microbiome in neurodevelopmental disorders suggests that therapeutic strategies targeting the microbiome could offer new avenues for treatment.^26^

As we gain more insight into the intricate relationship between the gut microbiome and pediatric health, it becomes increasingly clear that early-life microbial exposures and disruptions can have lasting effects on the development of various diseases. Understanding the factors that influence microbiome development during infancy and the mechanisms through which the microbiome impacts health and disease will be critical in developing targeted interventions to promote health. Probiotics, prebiotics, and other microbiome-based therapies are emerging as promising tools to modulate the microbiome and prevent or treat pediatric diseases. Fecal microbiota transplantation (FMT), for example, which has shown potential in treating recurrent *Clostridiodes difficile* infections and is being explored for conditions like IBD and autism.^27^

In this review, we adopt a hypothesis-driven framework in which early-life intestinal barrier permeability, microbe metabolite immune crosstalk, and environmental stressors interact to shape pediatric health outcomes. We synthesize evidence from human observational and interventional studies to examine how disruptions during critical developmental windows may predispose children to immune-mediated and neurodevelopmental disorders. Finally, we discuss emerging microbiome-targeted interventions including probiotics, prebiotics, and fecal microbiota transplantation and emphasize the need for longitudinal, mechanistic studies to determine whether early modulation of the microbiome can meaningfully alter disease trajectories.

## 2. Developmental assembly and functional maturation of the infant intestinal microbiota

The neonatal gut microbiome emerges from a relatively simple microbial community into a functionally complex ecosystem through coordinated taxonomic succession and metabolic maturation, progressively aligning with host nutritional demands and immune development. The comparatively simple neonatal microbiome develops during the first year after birth to a complexities organization that resembles the GI tract of an adult, with an increase in Firmicutes and Bacteroides.^28^ ^,^ ^29^ During the first year of life, the infant gut microbiome undergoes coordinated taxonomic and functional maturation, progressively converging toward the maternal gut metagenomic profile, with a concomitant reduction in inter-individual functional heterogeneity.^30^ The microbiome gets ready for an adult diet even before solid foods are introduced by gaining more bacterial genes related to the breakdown of plant polysaccharides.^31^ A discernible shift in the microbial composition occurs with the introduction of solid foods, with Bacteroidetes becoming more common. Higher levels of short-chain fatty acids in feces and increased expression of genes related to vitamin biosynthesis, xenobiotic degradation, and carbohydrate metabolism are additional changes.^32^

The early development of the gut microbiota is influenced by a number of variables. A long-term birth cohort study followed over 16,000 children for more than 20 years to identify early-life factors associated with neurodevelopmental disorders. Using longitudinal questionnaires and multiple infant biomarkers, the study found significant links between future ND diagnosis and early infections, antibiotic exposure, stress, immune and metabolic alterations, and microbiome changes. These associations spanned several ND subtypes, highlighting opportunities for early prediction and intervention.^33^ For instance, using antibiotics during this crucial time can drastically change the microbiome's structure.^16^ ^,^ ^29^ ^,^ ^32^ The development of the microbiome can also be significantly impacted by exposure to less sterile settings, including interactions with siblings and pets.^2^ ^,^ ^34^ In fact, having older siblings is associated with greater bacterial diversity and richness at 18 months, particularly with higher abundances of Firmicutes and Bacteroidetes in infants with more siblings.^35^ ^,^ ^36^

Additionally, the microbiome plays a crucial role in the health of infants and children. A longitudinal study of Malawian twins, one of whom suffered from Kwashiorkor, revealed that the malnourished twin had abnormal microbiome profiles compared to the healthy twin. To demonstrate that the microbiome contributed to the development of the Kwashiorkor phenotype, researchers transplanted frozen fecal samples from the twins into gnotobiotic mice.

**Figure 1.**
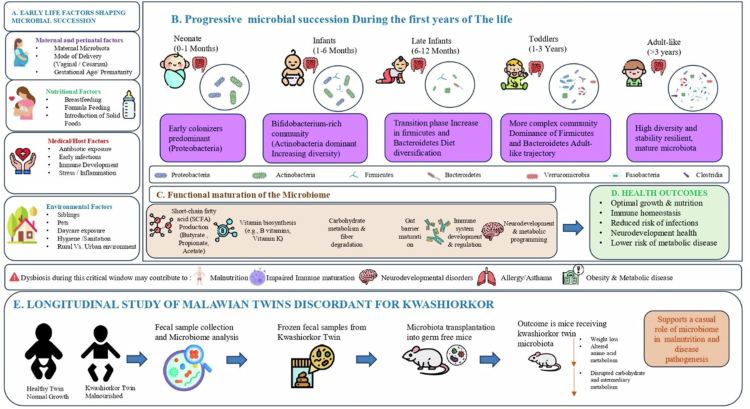
Developmental Assembly and Functional Maturation of the Infant Gut Microbiota and Its Implications for Health Outcomes. Created using https://BioRender.com.

Mice receiving the microbiome from the kwashiorkor twin experienced significant weight loss and disruptions in amino acid, carbohydrate, and intermediary metabolism.^36^ ^,^ ^37^ The infant gut microbiota undergoes dynamic taxonomic and functional maturation during early life, evolving from a relatively simple microbial community into a highly diverse and stable adult-like ecosystem. Initial colonizers, predominantly Proteobacteria and Actinobacteria, are progressively supplanted by increasing proportions of Firmicutes and Bacteroidetes as dietary diversification and environmental exposures occur. This microbial succession is strongly influenced by several early-life determinants, including mode of delivery, breastfeeding practices, maternal microbiota, antibiotic exposure, infections, sibling and pet interactions, and surrounding environmental conditions. Concurrently, the developing microbiome acquires enhanced functional capabilities related to short-chain fatty acid production, vitamin biosynthesis, carbohydrate metabolism, and immune system maturation. Disruptions to this critical developmental process may result in dysbiosis, potentially contributing to malnutrition, impaired immune development, neurodevelopmental abnormalities, and metabolic disorders. The lower panel illustrates findings from longitudinal studies of Malawian twins discordant for kwashiorkor, where transplantation of microbiota derived from malnourished infants into gnotobiotic mice induced weight loss and metabolic dysfunction, thereby supporting a causal role of the gut microbiome in disease pathogenesis (Figure 1).

Prebiotics are dietary compounds that promote host health by selectively stimulating beneficial gut microorganisms. While non-digestible oligosaccharides are the most widely studied prebiotics, emerging evidence suggests that amino acids (AAs) may also possess prebiotic properties. Certain AAs can modulate gut microbial composition, serve as selective substrates for beneficial bacteria, and generate bioactive metabolites that positively influence host metabolism and immune function. Because microbial AA metabolism is species- and strain-specific, AAs may help promote beneficial microbes while limiting pathogen growth. This has led to the introduction of the term “Aminobiotics” to describe amino acids with prebiotic-like functions. Studies also indicate synergistic effects between AAs and probiotic bacteria, supporting their potential use in novel synbiotic formulations. However, targeted delivery of free AAs to the distal gut remains challenging due to rapid absorption in the small intestine, highlighting the need for advanced delivery systems such as encapsulation. Further studies are needed to determine the optimal amino acids, dosages, and delivery strategies for improving human and animal health [Beaumont M, Roura E, Lambert W, Turni C, Michiels J, Chalvon-Demersay T. Selective nourishing of gut microbiota with amino acids: A novel prebiotic approach? Front Nutr. 2022 Dec 19;9:1066898].

## 3. Early microbial colonization: prenatal, perinatal, and environmental interactions

Early microbial colonization reflects the combined influence of prenatal exposures, birth-related microbial transfer, and postnatal environmental inputs, collectively shaping the initial host microbe interface during a period of heightened developmental plasticity. For a long time, it was thought that the fetal environment in the womb was completely sterile and that gut colonization started only after birth. However, recent research has identified microbial communities in meconium, challenging this long-held belief.^38^ ^,^ ^39^ While the topic remains under debate, emerging evidence indicates that microbial colonization of the infant gut might commence even before birth. Studies have detected the presence of microbes in the placenta.^21^ ^,^ ^40^ ^,^ ^41^, amniotic fluid^42^ ^,^ ^43^ and umbilical cord^44^ indicating possible prenatal exposure to microbes.

Aagaard et al.^40^ analyzed 230 sterile placental samples, revealing a unique placental microbiome similar to that of the human oral cavity. Furthermore, a randomized, double-blind, placebo-controlled study demonstrated that maternal probiotic supplementation can modulate the expression of Toll-like receptor (TLR)-associated genes in both the placenta and the fetal intestine.^43^ Interestingly, differences in the placental microbiome have been associated with preterm birth and with low birth weight among full-term infants.^21^

Beyond prenatal influences, multiple factors affect early gut colonization, such as the gestational age at delivery. Studies have demonstrated that the microbiota of preterm infants^45^ differs markedly from that of full-term infants, with preterm microbiomes being dominated by genera such as *Enterobacter*, *Enterococcus* and *Staphylococcus* ^46^ ^,^ ^47^ Premature birth is linked to an increased risk of neonatal complications, resulting in substantial morbidity and mortality.^48^ Preterm infants often experience extended hospital stays, antibiotic treatments, and formula feeding, all of which can disrupt the development of beneficial microbial communities. Changes in the microbiome of preterm infants have been associated with an increased risk of complications such as necrotizing enterocolitis and late-onset sepsis.

Diet also plays a key role in shaping the infant gut microbiome. Breastfed babies typically exhibit microbiota rich in *Lactobacillus*, *Staphylococcus*, and *Bifidobacterium*, whereas formula-fed infants tend to have a higher abundance of *Roseburia*, *Clostridium*, and *Anaerostipes* ^28^ Formula-fed infants also exhibit higher levels of microbes linked to inflammation and experience a more rapid shift toward an adult-type microbiome composition.^15^ ^,^ ^28^ ^,^ ^49^ ^,^ ^50^

On the other hand, studies suggest that human milk contains beneficial and potentially probiotic microbes.^28^ ^,^ ^49^ Human milk oligosaccharides (HMOs), which are the third-largest component of human milk, are prebiotic and possess antimicrobial and antiadhesive properties that may help protect the infant. Interestingly, breastfed infants generally have lower microbial diversity compared to formula-fed infants. However, this reduced diversity is associated with an increase in genes related to HMO degradation, which enhances the presence of specific bacterial populations in the infant gut.^36,51–54^

## 4. Early-life gut microbiome formation: interactions between host immunity, barrier maturation, and environment

**Figure 2.**
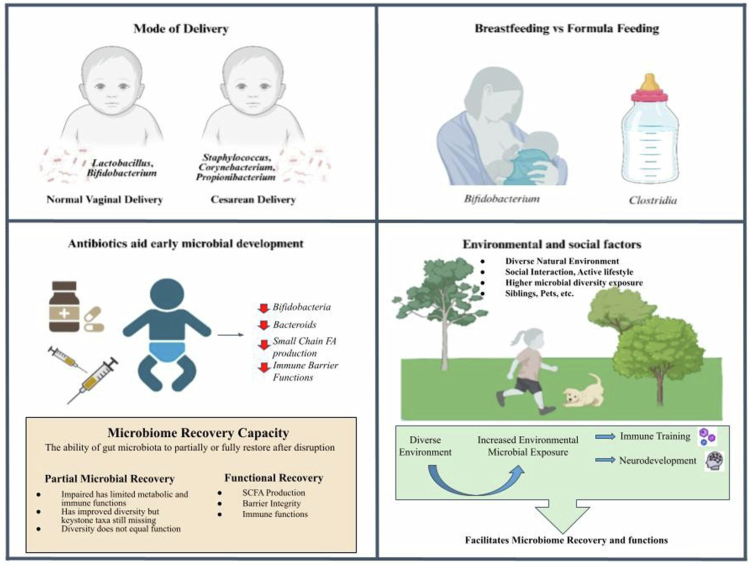
Factors influencing the pediatric gut microbe. The figure illustrates major early-life factors that shape the pediatric gut microbiome, including mode of delivery, feeding practices, antibiotic exposure, and environmental interactions. Vaginal delivery and breastfeeding support beneficial microbial colonization, whereas cesarean delivery, formula feeding, and antibiotics may disrupt microbial diversity and immune development. Environmental exposures such as siblings, pets, and natural surroundings contribute to microbiome resilience, immune maturation, and long-term health outcomes. Created using https://BioRender.com.

During the early years of life, the intestinal microbiome of children varies significantly due to a number of genetic and environmental factors. Immune system architecture and functions are either undeveloped or poorly adapted at birth compared to adulthood.^55^ ^,^ ^56^ In contrast to adults, neonatal neutrophils, monocytes, and dendritic cells exhibit diminished ability to combat and eliminate pathogenic infections.^57-59^ Moreover, the intestinal barrier in neonates remains highly permeable for up to six months, permitting the passage of ingested macromolecules, such as immunoglobulins (Igs), across the immature intestinal epithelium.^56^ ^,^ ^60^ Functional maturation of T and B lymphocytes begins approximately 12 weeks postnatally; however, during this early developmental stage, these cells are more inclined to promote antigen tolerance rather than initiate effective immune clearance.^61-63^ During the early weeks and months of life, the infant gut microbiome matures rapidly, playing a vital role in immune system development. It contributes to defense against pathogenic microbes, supports the maturation of mucosal immune structures, and facilitates the establishment of antigen tolerance.^64^ Understanding how different sources of early nutrition particularly human milk and human milk substitutes of different formulations affect gut microbiome development and immune system maturation is essential because disturbances during this period may have long-lasting health consequences^65^ (Figure 2).

### 4.1. An integrated framework linking barrier function, microbial signaling, and environmental exposures

Accumulating evidence supports a model in which early-life health trajectories are governed by the intersection of intestinal barrier permeability, microbe-derived metabolite immune crosstalk, and environmental stressors during defined developmental windows. Relative immaturity of the epithelial barrier permits increased translocation of microbial products and metabolites, including short-chain fatty acids, bile acid derivatives, tryptophan metabolites, and microbial associated molecular patterns, thereby influencing immune education and systemic inflammatory tone^66^ These signals integrate with innate and adaptive immune pathways to shape immune tolerance, neuroimmune communication, and metabolic programming^67^ Short Chain Fatty Acid (SFCA)-producing bacteria, mucin degraders, bile acid–modifying microbes are implicated in epithelial maintenance and permeability regulation. Environmental stressors, such as antibiotic exposure, dietary contaminants, psychosocial stress, and infection, can exacerbate barrier dysfunction and modify microbial metabolic output, shifting host microbiome interactions from homeostatic to dysregulated states. Disruption of this interconnected network provides a plausible mechanistic link between early-life exposures and increased susceptibility to immune-mediated, metabolic, and neurodevelopmental disorders. There is a need for functional and longitudinal human studies to move beyond taxonomic associations.

### 4.2. Balanced immune signaling, nutrition, and barrier integrity

Balanced immune signaling is essential for the establishment and maintenance of host microbiome homeostasis during early life, and nutrition plays a central role in coordinating immune maturation, microbiome development, and intestinal barrier integrity. Dietary inputs shape microbial succession and metabolic activity, influencing the production of short-chain fatty acids, indole derivatives, and other metabolites that reinforce epithelial tight junctions and regulate mucosal immune responses. In parallel, bioactive components of human milk, including HMOs,^36^ ^,^ ^51-54^ immunoglobulins, and antimicrobial peptides, directly support barrier maturation and immune tolerance while selectively promoting beneficial microbial taxa. Disruption of early nutritional signals, whether through suboptimal feeding practices or premature dietary transitions, can impair barrier function and skew immune signaling towards inflammatory phenotypes, thereby increasing vulnerability to infection and immune-mediated disease^68^ Together, coordinated nutritional and immune cues are critical for sustaining epithelial integrity and balanced host–microbiome interactions during this developental window.

### 4.3. Mode of delivery and initial host–microbe interactions

Mode of delivery represents one of the earliest determinants of host microbe interaction, influencing the composition of pioneer microbial communities and the initial immune cues encountered by the neonate. The mode of birth delivery has a profound effect on the initial microbiome composition. Infants born vaginally are exposed to their mother's microbiota, particularly from the birth canal, which leads to early colonization by *Lactobacillus*, *Bifidobacterium*, and other protective microbes. In contrast, infants delivered by cesarean section acquire microbiota from the maternal skin and hospital environment, resulting in a microbiome dominated by *Staphylococcus*, *Corynebacterium*, and *Propionibacterium* species. Studies have demonstrated that cesarean-born infants are more likely to develop conditions such as asthma and obesity later in life.^69^ Between 1977–1978 and 2017–2018, whole-grain consumption remained substantially lower than refined-grain intake, despite longstanding federal dietary recommendations introduced in 2005 advising that at least half of grain intake should come from whole grains. Across this period, adults aged 65 years and older consistently consumed diets with higher whole-grain density compared with younger adults and children. Notably, a meaningful increase in whole-grain density was observed only in children’s diets during 2013–2018 relative to 1994–2010, coinciding with the implementation of updated school meal nutrition standards that mandated regular inclusion of whole-grain-rich foods.^70^

### 4.4. Early nutrition as a driver of microbial–immune co-development

Early nutrition acts as a primary regulator of microbial succession and immune co-development, with human milk providing both microbial substrates and bioactive signals that shape mucosal immunity. Breastfeeding plays a vital role in shaping the infant's gut microbiome, largely due to the prebiotics and probiotics found in human breast milk.^71^ Breast milk is rich in human milk oligosaccharides (HMOs), which act as prebiotics that support the growth of beneficial bacteria such as *Bifidobacteria*.^72^ Prebiotics are food components that are not digested by human enzymes but are metabolized by specific bacteria, promoting their growth and contributing to the health benefits of the host.^71^ Breastfeeding has been linked to increased microbiome diversity and enhanced immune function.^72^ In contrast, formula-fed infants tend to have a less diverse microbiome, often dominated by *Clostridia* and *Enterobacteriaceae*, which can increase the risk of inflammatory diseases and infections.^2^

### 4.5. Antibiotic exposure as a disruptor of microbial and immune network maturation

Antibiotic exposure during early life perturbs the developing microbial ecosystem, with significant downstream consequences for immune education, metabolic regulation, and long-term disease susceptibility. The incidence of infections remains high during the neonatal period, and several neonatal conditions require the administration of broad-spectrum antibiotics. However, antibiotic therapy administered before, during, or after birth can adversely affect the neonatal microbiota and subsequently influence the maturation of the infant immune system.^71^ Early-life antibiotic exposure disrupts the gut microbiome by reducing microbial diversity,^73^ ^,^ ^74^ decreasing the abundance of beneficial bacteria such as Bifidobacteria and Bacteroidetes,^71^ and promoting the expansion of potentially pathogenic microorganisms. Such microbial alterations may impair key metabolic pathways, including bile acid biotransformation and short-chain fatty acid production, thereby affecting host metabolism, immune signaling, and nutrient absorption. In addition, antibiotic-induced dysbiosis may alter mucin dynamics by reducing mucin-degrading and butyrate-producing bacteria, potentially compromising mucus layer integrity and intestinal barrier function. Early antibiotic exposure has also been associated with an increased risk of asthma, allergies, obesity, and other atopic diseases.^71^ ^,^ ^73^ ^,^ ^74^ The timing, duration, and frequency of antibiotic administration may exert long-lasting effects on microbiome composition and functional maturation, with some studies suggesting that these alterations may persist into later life.^73^ ^,^ ^74^

### 4.6. Environmental and social modulators of microbial diversity and immune education

Environmental and social exposures introduce microbial diversity and antigenic complexity that influence immune tolerance and microbiome stability during early childhood. The environment, including factors such as geographic location, hygiene practices, exposure to pets, siblings, and outdoor environments, can influence the development of the pediatric gut microbiome. For example, children raised on farms or in rural environments have more diverse microbiomes compared to urban children, possibly contributing to a lower risk of allergic diseases.^75^ Additionally, early exposure to diverse microbiota may strengthen immune tolerance and reduce the risk of autoimmune diseases.

Early-life nutrition plays a pivotal role in regulating microbial succession and immune maturation, thereby shaping the development of a stable and functionally diverse gut microbiome. In addition to breastfeeding, several dietary exposures including formula feeding, timing of complementary food introduction, dietary diversity, micronutrient supplementation, and the intake of fibers, whole grains, and protein-rich foods significantly influence microbial–immune co-development during infancy. Human milk contains bioactive compounds such as human milk oligosaccharides (HMOs), immunoglobulins, and beneficial microbes that selectively promote the growth of Bifidobacteria and support mucosal immune homeostasis.

[Zhang B, Li LQ, Liu F, Wu JY. Human milk oligosaccharides and infant gut microbiota: Molecular structures, utilization strategies and immune function. Carbohydr Polym. 2022 Jan 15;276:118738. doi: 10.1016/j.carbpol.2021.118738.; Dinleyici M, Barbieur J, Dinleyici EC, Vandenplas Y. Functional effects of human milk oligosaccharides (HMOs). Gut Microbes. 2023 Jan-Dec;15(1):2186115. doi: 10.1080/19490976.2023.2186115]. The introduction of complementary foods further enhances microbial diversity, with increased abundances of Firmicutes and Bacteroidetes and greater metabolic activity related to carbohydrate fermentation and short-chain fatty acid production.

Dietary proteins and plant-derived nutrients also contribute to microbial metabolite generation, intestinal barrier integrity, and immune regulation. Conversely, inadequate nutrition, delayed or inappropriate complementary feeding, and reduced dietary diversity may impair microbiome maturation and increase susceptibility to infections, inflammatory disorders, metabolic dysfunction, and adverse neurodevelopmental outcomes. Relevant supporting studies have demonstrated the role of HMOs in promoting Bifidobacterium-dominant microbiota and immune development, as well as the impact of complementary feeding and dietary diversity on infant gut microbial maturation.

## 5. Continued functional refinement of the gut microbiome across childhood and adolescence

Beyond infancy, the gut microbiome continues to undergo functional refinement, reflecting ongoing interactions between host growth, dietary diversification, immune maturation, and environmental exposures. Some studies suggest that the pediatric gut microbiome reaches an adult-like composition within the first three years of life.^4^ ^,^ ^76^ while other studies suggest that its development continues into childhood and adolescence.^77-79^ A comparative analysis of intestinal microbiota between children aged 1–4 years and healthy adults showed that adults possess considerably higher microbial diversity in terms of both abundance and richness. While the dominant bacterial phyla *Firmicutes*, *Bacteroidetes*, and *Actinobacteria* were common to both groups, notable variations were observed at the genus level.^78^

A birth cohort study supports the hygiene hypothesis by showing distinct early gut microbiome patterns across Northern Europe, with Bacteroides dominating in regions with higher autoimmune disease incidence. Structurally distinct Bacteroides LPS dampened innate immune signaling, suggesting that early exposure to immunologically “silent” microbes may impair immune education and increase autoimmune risk^80^ Research comparing the fecal microbiota of adolescents (ages 11–18) and healthy adults revealed a similar number of microbial species, yet variations in the relative abundance of several genera indicated that the adolescent gut microbiome is still distinct from the adult profile.^77^ Hollister et al.^68^ analyzed the gut microbiota of children aged 7–12 years and adults, finding that the pediatric microbiome, similar to that of adults, was primarily dominated by *Bacteroidetes* and *Firmicutes*. However, the proportions of these bacterial groups varied between the two age groups. Children exhibited lower levels of *Bacteroidetes* but higher levels of *Firmicutes* and *Actinobacteria*. Additionally, although many taxa were common to both pediatric and adult samples, children showed significantly greater abundances of genera such as *Faecalibacterium*, *Dialister*, *Roseburia*, *Ruminococcus*, and *Bifidobacterium*. Notably, Hollister et al. analyzed the metagenomic profiles of pediatric and adult microbiomes and found that children showed an enrichment of genes associated with growth and development, such as those involved in vitamin production, *de novo* folate synthesis, and amino acid metabolism. In contrast, adult microbiomes were enriched with pathways related to inflammatory processes, including genes involved in oxidative phosphorylation, lipopolysaccharide biosynthesis, flagellar assembly, and steroid hormone synthesis. Although the intestinal microbial communities of children showed only 35–46% similarity in taxonomic composition, they demonstrated over 90% similarity at the ortholog group and metabolic pathway levels. This indicates that the functional potential of microbes in the pediatric gut is more conserved than their overall taxonomic composition.^36^

## 6. Gut virome development and its integration with bacterial and host immune networks

The gut virome develops alongside bacterial communities and host immunity, forming an interconnected network in which bacteriophages and eukaryotic viruses modulate microbial composition and immune homeostasis. The process of gut virome development begins at birth, marking a critical transition from a sterile in utero environment to one rich in microbial and viral exposures. Viral colonization commences almost immediately after delivery whether by cesarean section (CS) or vaginal delivery (VD) with the mode of birth influencing the diversity of viral and bacteriophage communities that establish early in life.^81^ Unlike the bacterial microbiome, the gut virome remains less understood, particularly in terms of its origin, composition, and the various factors that influence its establishment (Figure 1). While culture-based and PCR techniques have revealed the presence of specific eukaryotic viruses in infant gut samples, only a limited number of comprehensive metagenomic studies have been conducted. These studies indicate that the infant gut hosts a diverse array of bacteriophages, and that eukaryotic viruses are present sporadically.^22^

Evidence suggests that the newborn gut is largely virus-free at birth and rapidly colonized by bacteriophages within the first week of life. These early phages likely originate from induced prophages of pioneering gut bacteria acquired through the birth canal, breast milk, or the maternal gut microbiota. For instance, prophages carried by certain *Bifidobacterium* species can be transmitted via breast milk and subsequently induced in the infant gut. Unlike the bacterial community, which increases in diversity during early development, the initially phage-rich virome declines in richness over time, stabilizing into an adult-like composition by around 2–3 years of age.^82^

### 6.1. Age-dependent composition and individualization of the gut virome

Gut virome composition is highly individualized and age-dependent, shaped by early-life exposures, host immunity, and interactions with resident bacterial populations. The human virome exhibits a site-specific composition across various anatomical compartments, including the blood, GI (GI) tract, respiratory tract, urogenital system, and skin. Among these, the GI tract hosts the largest concentration of viruses, with an estimated 10^9^ to 10^10^ viral-like particles (VLPs) per gram of feces. Research, including studies by our team and others, has consistently shown that the gut virome is highly individualized, varying based on factors such as geography, lifestyle, diet, and age.^83^ Early feeding methods play a crucial role in shaping the human gut virome. Both eukaryotic viruses and bacteriophages are transferred from mother to infant through breastfeeding, which leads to variations in the composition of the infant's gut virome, depending on whether they are breastfed or formula-fed. This difference is partly attributed to the maternal antibodies passed through breastmilk, providing additional immunity that is not transmitted through formula milk. The mode of birth also creates distinguishable differences in virome composition. Babies born via vaginal delivery (VD) exhibit a more diverse virome compared to those born via cesarean section (CS). In particular, viruses such as *Caudoviricetes, Microviridae, and Anelloviridae* are among the most abundant in infants delivered by VD.^81^

### 6.2. Determinants of gut virome assembly and stability

The assembly and stability of the gut virome are governed by a convergence of prenatal exposure, host genetics, immune competence, diet, and close interpersonal contact.

#### 6.2.1. Prenatal exposure

Certain pathogenic eukaryotic viruses including HIV, influenza, rubella, varicella, cytomegalovirus (CMV), and human papillomavirus (HPV) can be transmitted to the fetus either transplacentally or during vaginal delivery. Clinical outcomes depend on the specific virus and the gestational age at exposure, and may range from miscarriage to asymptomatic infection.^22^

#### 6.2.2. Inter individual variability

Although the gut virome is characterized by substantial interindividual variability, studies in twins provide a unique context in which this variability is constrained.^84^ ^,^ ^85^ During infancy, gut viromes are significantly more similar between twin pairs than between unrelated infants, consistent with evidence of frequent transmission of vaccine-strain rotavirus from vaccinated infants to their unvaccinated co-twins, implicating close physical contact as a key driver of virome sharing. Notably, the viromes of infant co-twins remain distinct from those of their mother or older non-twin siblings, highlighting age-dependent host and environmental factors as major determinants of early-life viral community composition. The influence of genetic factors on the infant gut virome remains largely unclear.^22^

#### 6.2.3. Dietary influence

While diet is a well-established modulator of the bacterial microbiome, its role in shaping the gut virome is less defined. A study by Minot et al. followed six healthy adults during an 8-day dietary intervention. Participants on the same diet developed more similar viromes by the end of the intervention, despite each individual maintaining a unique and largely stable virome throughout the period. These findings suggest that while food is unlikely to be a major direct source of phage populations, dietary composition does influence the virome. Notably, shifts in dietary components such as fat, gluten, or alcohol can induce subtle but measurable changes in virome composition.^81^ Thus, although inter-individual variability is the primary determinant of virome diversity, shared dietary patterns may lead to convergent virome profiles.

#### 6.2.4. Host genetics and immune response

The host’s genetic background and immune status are key determinants of gut virome composition. Variations in genes regulating pattern recognition receptors can alter antiviral responses, influencing how effectively enteric viruses are detected and cleared. Under healthy conditions, innate and mucosal immunity maintain balance: type I and III interferons restrict viral replication, secretory IgA limits viral entry into epithelial cells, and antigen-presenting cells activate T cells, triggering cytokines such as IL-22, IL-15, and interferons that further shape virome dynamics. When immunity is compromised, as in HIV-associated CD4⁺ T cell depletion within gut-associated lymphoid tissue, this equilibrium breaks down, enabling opportunistic viruses like adenovirus and CMV to proliferate unchecked.^86^

## 7. Prenatal determinants of fetal microbial exposure and early immune programming

Prenatal factors play a foundational role in shaping early microbial exposure and immune programming by influencing maternal microbial communities and modulating inflammatory, metabolic, and hormonal signals at the maternal fetal interface. Although direct fetal colonization remains debated, accumulating evidence indicates that maternal microbiota derived metabolites, microbial components, and immune mediators reach the fetus via the placenta and amniotic fluid, priming neonatal immune responses before birth. Disruptions during pregnancy can therefore alter immune set points and microbial trajectories with lasting consequences for postnatal microbiome assembly and disease susceptibility.

### 7.1. Maternal factors during pregnancy

Maternal exposures during pregnancy, particularly antibiotic use and metabolic status, exert strong selective pressures on maternal microbial communities, with downstream effects on neonatal microbial acquisition and immune development. Antibiotic exposure during gestation can disrupt maternal gut and vaginal microbiota, often increasing colonization by opportunistic taxa such as Staphylococcus while reducing beneficial anaerobes.^87^ These alterations can influence the microbial inoculum encountered by the infant at birth and have been associated with increased risks of allergic disease, otitis media, and childhood obesity. In parallel, maternal metabolic states, including insulin resistance^88^ and low-grade inflammation, can modify microbial metabolite profiles and immune signaling, potentially biasing fetal immune development toward pro-inflammatory phenotypes. Collectively, these findings suggest that maternal microbiome perturbations during pregnancy may indirectly shape infant immune trajectories through both microbial and immunological pathways.

### 7.2. Influence of gestational age

Gestational age represents a critical modifier of prenatal microbial and immune exposure, with prematurity associated with both infectious and inflammatory intrauterine conditions. Ascending infections from the genitourinary tract can trigger inflammation, membrane rupture, and preterm birth, increasing fetal exposure to microbial products in utero.^71^ Preterm infants exhibit marked immunological immaturity, characterized by reduced leukocyte numbers, attenuated pro-inflammatory cytokine responses, and diminished antimicrobial peptide production^89^ In addition, prematurity is frequently accompanied by obstetric interventions, including cesarean delivery and prenatal antibiotic administration, further compounding microbial disruption. Even under optimal postnatal conditions such as vaginal delivery, breastfeeding, and limited antibiotic use, the gut microbiome of preterm infants remains distinct from that of term infants, reflecting both altered prenatal exposures and intrinsic developmental constraints on immune–microbiome co-maturation.^90^

### 7.3. Maternal obesity

Obesity is associated with an imbalance in the Firmicutes-to-Bacteroidetes ratio in the gut. Infants born by C-section to overweight mothers have a higher risk of becoming overweight later in life compared to those born vaginally to overweight or obese mothers. Maternal weight status also affects breast milk composition, with obese mothers showing higher bacterial counts, as well as increased levels of *Staphylococcus* and *Lactobacillus*, and reduced *Bifidobacterium* in their milk during the first six months of lactation.^91^

### 7.4. Diet and nutrition during pregnancy

Maternal diet represents a modifiable determinant of the maternal microbiome and its metabolic outputs, with implications for fetal immune priming and neonatal microbiome assembly. Dietary patterns rich in fat have been associated with shifts toward higher Firmicutes-to-Bacteroidetes ratios, whereas increased dietary fiber intake promotes microbial diversity and short-chain fatty acid production, which can modulate immune tolerance pathways. Consumption of probiotic-containing foods during pregnancy has been linked to reduced risk of spontaneous preterm birth and lower incidence of allergic disease in offspring, although findings remain heterogeneous. Micronutrients such as vitamin D have also been shown to influence maternal microbplant dietial composition, with some studies reporting reduced alpha diversity and enrichment of Actinobacteria and Proteobacteria. Additionally, maternal diet-driven changes in taxa such as *Prevotella* may influence infant susceptibility to food allergies. Together, these observations underscore the role of prenatal nutrition in shaping microbial–immune signaling pathways relevant to long-term pediatric health.^92^ ^,^ ^93^

## 8. Gut–brain axis development: microbial metabolites, neuroimmune signaling, and brain maturation

Early brain development occurs in parallel with rapid microbial and immune maturation, with microbial metabolites and immune mediators acting as key signals linking the gut to neurodevelopmental processes. During the perinatal period, the human brain experiences a phase of rapid growth that coincides with profound shifts in the maternal gut microbiota. As pregnancy advances from the first to the third trimester, there is a marked increase in Proteobacteria and Actinobacteria, along with a decline in overall microbial diversity.^94^ Although these microbial changes resemble those observed in metabolic syndrome in non-pregnant individuals, they play an adaptive role during pregnancy, promoting energy storage and supporting fetal development.

**Figure 3.**
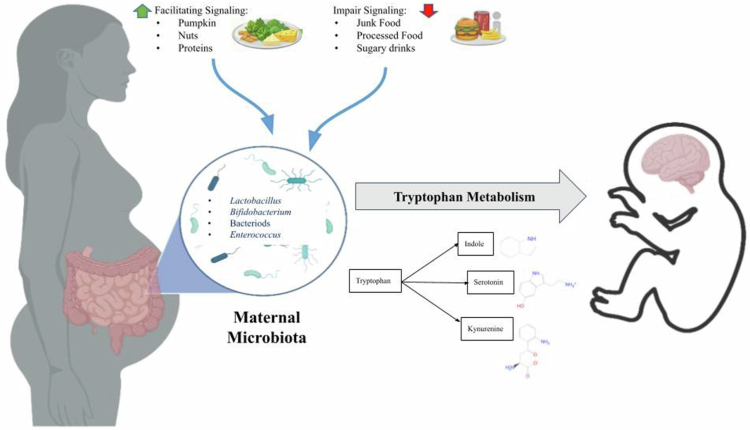
Maternal Microbiota-Mediated Modulation of Fetal Brain Development via Tryptophan-Serotonin Pathways. The figure illustrates the influence of maternal diet and gut microbiota on fetal neurodevelopment through tryptophan metabolism. Beneficial dietary factors promote microbial populations involved in the production of neuroactive metabolites such as serotonin, indole, and kynurenine, whereas unhealthy dietary patterns may impair these pathways. Maternal microbial metabolites can modulate signaling along the gut–brain axis, thereby influencing fetal brain development and neurodevelopmental outcomes. Created using https://BioRender.com.

Emerging evidence highlights the critical influence of the gut microbiota on early brain development, particularly through its modulation of tryptophan metabolism and serotonin (5-HT) synthesis, which are both essential for the maturation of the central nervous system (CNS).^95^ (Figure 3). Mouse studies involving mutations in the tryptophan hydroxylase 2 gene, an enzyme vital for 5-HT synthesis, have demonstrated that deficient brain serotonin can lead to aberrant neural wiring, increasing the risk of neurodevelopmental disorders.

Comparative research between germ-free (GF) and specific pathogen-free (SPF) mice further supports this connection. GF mice display increased locomotor activity, reduced anxiety, and altered levels of neurotransmitters including dopamine, noradrenaline, and 5-HT deficits that can be reversed through early-life exposure to microbiota.^96^

GF mice also exhibit a lower kynurenine/tryptophan ratio compared to their SPF counterparts, a condition that normalizes following gut microbial colonization after weaning.^97^ This microbial exposure is also essential in restoring normal anxiety responses. In another study, Bifidobacterium infantis-treated rats showed decreased levels of 5-HIAA (a serotonin metabolite) in the frontal cortex, along with elevated plasma tryptophan and kynurenic acid levels compared to controls.^98^

Overall, gut microbiota plays a direct and multifaceted role in tryptophan metabolism and 5-HT biosynthesis. Some bacterial strains are capable of producing 5-HT from tryptophan, while others use tryptophan synthase or tryptophanase enzymes to synthesize or degrade it.^99-102^

Colonic bacteria are essential for fermenting carbohydrates and proteins, generating metabolites like SCFAs that are critical for human health. According to Erny et al., SCFAs help maintain microglial homeostasis, and disruptions in microbiota composition such as in germ-free or microbiota-deficient mice can lead to impaired microglial function.^103^ Early interaction with native microbiota is essential for the normal maturation of the hypothalamic-pituitary-adrenal (HPA) axis. Germ-free mice display an exaggerated HPA stress response, which can be normalized through early exposure to SPF fecal microbiota. However, introducing SPF fecal microbiota at a later developmental stage fails to correct the abnormal stress response. These findings highlight the critical role of microbiota in early postnatal brain development and suggest that some degree of neural plasticity may still persist.^36^

### 8.1. Bidirectional microbiota–brain communication and neurocognitive outcomes

The gut-brain axis operates as a bidirectional communication system integrating neural, immune, endocrine, and microbial signals to regulate cognitive function and stress responsiveness. The brain–gut–microbiota axis comprises an intricate communication network linking the central nervous system (CNS), neuroendocrine and neuroimmune pathways, the sympathetic and parasympathetic divisions of the autonomic nervous system, the enteric nervous system, and the intestinal microbiota. This two-way communication enables the brain to regulate the GI tract’s motor, sensory, and secretory activities, while signals originating from the gut, in turn, influence brain function.

Prolonged administration of the probiotic *Lactobacillus rhamnosus* (JB-1) has been reported to reduce stress-induced corticosteroid levels, alleviate depressive behaviors, and decrease anxiety. Additionally, *L. rhamnosus* treatment caused region-specific alterations in the expression of GABAB1b and GABAAα2 receptors both associated with anxiety and depression. Interestingly, these effects were not observed in vagotomized mice, highlighting the vagus nerve as a key communication route between gut microbes and the brain.^103^

A separate study identified a relationship between the gut microbiome and pain modulation, demonstrating that specific *Lactobacillus* strains stimulate the expression of *μ*-opioid and cannabinoid receptors in intestinal epithelial cells, effectively replicating morphine-like analgesic effect.^104^

The brain is also capable of modulating the gut microbiota’s composition. This influence may occur indirectly through alterations in gut motility and secretion, or directly via signaling molecules released into the GI tract by enterochromaffin cells, neurons, and immune cells.^105^ The autonomic nervous system (ANS) regulates gut motility and mucus secretion, thereby influencing the GI environment and, in turn, altering the composition of the resident microbial community.^106^ ^,^ ^107^ Furthermore, the ANS can modulate epithelial processes involved in gut immune activation. For instance, exposure to stress may elevate epithelial permeability, permitting bacterial antigens to penetrate and stimulate a mucosal immune response, consequently disrupting the microbiome.^107^ This heightened permeability is linked to mast cell degranulation, excessive production of interferon-*γ*, and decreased expression of mRNA for tight junction proteins.^36^ ^,^ ^108^

### 8.2. Dietary modalities and food-borne pollutants: a critical but understudied determinant of immune and neurodevelopment

Dietary modalities and exposure to food-borne pollutants represent important yet underexplored determinants of early-life microbiome–host interaction with direct relevance for immune signaling and brain development. Nutrient composition and dietary patterns profoundly shape microbial metabolism, modulating the production of short-chain fatty acids and neuroactive metabolites that influence intestinal barrier function, systemic immunity, and gut–brain communication pathways critical for neurodevelopment.^109^ ^,^ ^110^ However, research to date has largely focused on macronutrients and probiotic supplementation, with limited integrated study of how processed food components and contaminants interact with microbiota immune neural signaling. In parallel, food-borne pollutants such as heavy metals,^111^ pesticides,^112^ plasticizers, and other xenobiotics can perturb gut microbial communities, compromise barrier integrity, and disrupt bidirectional gut brain axis signaling, with emerging evidence linking such exposures to microbial dysbiosis and neurotoxicity in experimental and observational models. These exposures may be particularly impactful during developmental windows of immune and neural plasticity, yet the mechanisms by which diet quality and contaminant exposures intersect with microbiome-mediated pathways regulating immunity and brain development remain poorly defined, highlighting a critical knowledge gap that warrants mechanistic, longitudinal investigation.

## 9. Gut virome host interactions in intestinal and systemic disease

Alterations in gut virome composition can disrupt microbial equilibrium and immune regulation, contributing to both intestinal inflammation and systemic disease manifestations. Alterations in the gut virome are increasingly recognized as contributors to diseases such as *Clostridioides difficile* infection (CDI), inflammatory bowel disease (IBD), and colorectal cancer (CRC). The virome may drive pathology by directly modulating host immunity and by disrupting microbial balance. While bacteriophages play a major role, pathogenic enteric viruses such as norovirus and rotavirus have also been linked to IBD. Norovirus can alter host gene expression and trigger inflammation, with animal models showing accelerated disease in mice lacking immune regulators like IL-10 or ATG16L1. Rotavirus similarly promotes mucosal damage by increasing permeability, impairing epithelial renewal, and activating immune responses through mechanisms including NSP4 signaling and dendritic cell activation. In addition, viruses such as EBV and CMV may aggravate IBD in immunocompromised hosts, underscoring the broader impact of eukaryotic viruses on gut inflammation. Collectively, these findings highlight the disease-promoting potential of both phages and eukaryotic viruses, particularly in genetically susceptible individuals.^86^

## 10. Disruption of microbial–host homeostasis and pediatric disease susceptibility

Disruption of microbial–host homeostasis shifts symbiotic interactions toward pathogenic outcomes, increasing vulnerability to a spectrum of communicable and non-communicable pediatric diseases. Although a balanced microbiome is essential for maintaining overall health, disruptions in this equilibrium can shift the microbiome–host interaction from a symbiotic to a pathogenic state. The following sections highlight examples of both communicable and non-communicable diseases associated with microbiome imbalanced gut. Early-life gut microbiota plays a crucial role in immune maturation, and disruptions from factors such as antibiotics, cesarean delivery, or formula feeding are linked to increased risk of pediatric autoimmune diseases. Although microbiome-modulating interventions can partially restore dysbiosis, evidence for direct prevention of autoimmune disease is still lacking, underscoring the need for longitudinal studies.^113^ A birth cohort study investigated genetic, environmental, and infant gut microbiota factors associated with juvenile idiopathic arthritis (JIA). Children who later developed JIA showed distinct gut microbial alterations at one year of age, along with increased risk linked to shorter breastfeeding and higher antibiotic exposure, particularly in genetically predisposed individuals. The findings suggest that early-life microbial dysregulation and environmental exposures may contribute to JIA pathogenesis and offer potential avenues for early screening and intervention.^114^ ^,^ ^115^

### 10.1. *Clostridiodes difficile* infection

*C. difficile* infection (CDI) is the leading healthcare-associated infection in the U.S., affecting over 500,000 people annually.^116^ A healthy and diverse gut microbiota protects against CDI, while disruptions increase susceptibility. Schubert et al. reported that key bacterial families such as *Ruminococcaceae, Lachnospiraceae*, Bacteroides, and *Porphyromonadaceae* were depleted in CDI patients, especially in recurrent cases after repeated antibiotic use.^117^

Recurrent CDI is associated with an increase in Proteobacteria and a decrease in Bacteroides and Firmicutes compared to healthy individuals.^118^ Most gut microbiome research has traditionally concentrated on its bacterial constituents, but it is now clear that viruses also play a significant role. Recent investigations have demonstrated that transferring viral communities from healthy donors can resolve recurrent *Clostridioides difficile* infections, mitigate diet-induced obesity, and protect preterm infants from necrotizing enterocoliti*s*. Although the precise mechanisms remain to be elucidated, these benefits likely arise from the ability of bacteriophages the predominant viruses in the gut to reshape the bacterial microbiome through targeted infection.^119^

Antibiotic use is the primary risk factor for CDI, often leading to lasting changes in the microbiota.^120^ These changes typically reduce the taxonomic and functional diversity of the gut microbiome, impairing its ability to resist pathogenic colonization.^16^ Furthermore, Denève et al. found that exposure to subinhibitory concentrations of certain antibiotics can upregulate genes related to *C. difficile's* colonization factors, enhancing its adherence to cultured cells.^121^

The use of proton pump inhibitors (PPIs) also increases the risk of CDI.^122^ PPIs are known to decrease microbial diversity and reduce the abundance of beneficial gut microorganisms.^123^ Chronic PPI use leads to a shift in microbiota composition, with a decrease in Bacteroidetes and an increase in Firmicutes at the phylum level, potentially increasing susceptibility to CDI.^124^ Additionally, PPIs have been linked to higher fecal levels of *Enterococcaceae* and *Streptococcaceae*, which are associated with CDI.^125^

Therapies aimed at reestablishing healthy and diverse gut microbial communities have shown great promise in treating CDI. Fecal microbiota transplantation (FMT) achieves approximately a 90% cure rate for recurrent CDI after one or more treatments.^27^ The rationale behind FMT is that introducing a healthy fecal microbiome can restore both the structure and function of the gut microbial community, including colonization resistance.^126^ Weingarden et al. ^118^ demonstrated that FMT not only reestablishes bacterial diversity but also normalizes metabolic activity. Prior to FMT, fecal samples contained elevated levels of primary bile acids and bile salts, whereas post-FMT samples were enriched in secondary bile acids.^118^ Patients with recurrent CDI exhibited reduced conversion of primary to secondary bile acids a crucial process since primary bile acids promote *C. difficile* germination and growth, while secondary bile acids inhibit it. Thus, the therapeutic success of FMT may depend as much on restoring bile acid metabolism as on reconstituting the gut microbiota itself.^127^ ^,^ ^128^

### 10.2. Inflammatory bowel disease

Inflammatory bowel disease (IBD), including Crohn’s disease and ulcerative colitis, has been associated with an imbalanced or dysbiotic gut microbiome.^129^ ^,^ ^130^ Nevertheless, it remains unclear whether the microbial imbalance drives the disease, results from it, or represents a combination of both. Individuals with IBD often exhibit an excessive immune response to their gut microbes, which may play a key role in triggering intestinal inflammation.^131^ ^,^ ^132^ Conversely, some researchers propose that an altered gut microbiome acts as a major trigger for inflammation in IBD.^133^ In line with this view, studies have shown that antibiotic treatment can substantially lessen intestinal inflammation and relieve IBD symptoms. Furthermore, certain microbial species that are increased in IBD patients may contribute to the worsening of the condition.^134^ ^,^ ^135^ For instance, Ohkusa et al.^136^ ^,^ ^137^ observed a higher abundance of Fusobacterium species in patients with ulcerative colitis compared to healthy individuals. In a separate study, they demonstrated that introducing *Fusobacterium varium* via enema caused ulcerative colitis–like lesions in mice, suggesting its possible involvement in the disease’s development.^136^ ^,^ ^137^

Individuals with IBD also exhibit notable alterations in their fecal microbiota, characterized by reduced levels of *Bacteroides*, *Firmicutes*, *Clostridia*, *Bifidobacterium*, and *Lactobacillus*, along with increased levels of *Fusobacterium* and adherent-invasive *Escherichia coli*.^135^ ^,^ ^138^ ^,^ ^139^ Moreover, individuals with IBD exhibit decreased microbial diversity, especially within inflamed regions of the intestine compared to non-inflamed sites.^140^ The functional composition of the gut microbiota is also altered, with one study reporting that 12% of metabolic pathways were affected in IBD patients, compared to just 2% of genera when compared to healthy individuals.^10^ In IBD, functional changes include impaired carbohydrate metabolism and a decrease in the production of butyrate and other short-chain fatty acids.^10^ ^,^ ^141^

Considering the microbiome’s involvement in IBD, treatments aimed at modulating the gut microbiota have attracted interest. Some research indicates that certain antibiotic combinations may be beneficial for IBD management, although further controlled studies are required.^142^ Studies on the use of probiotics in IBD have shown encouraging results, but the findings have been inconsistent, underscoring the need for additional randomized controlled trials.^143–145^ Finally, fecal microbiota transplantation (FMT) holds potential as a therapeutic option for IBD, especially in light of its proven success in treating recurrent *C. difficile* infections.^146^ However, only a limited number of randomized controlled trials have focused on IBD, and their outcomes have been inconsistent, highlighting the need for more extensive studies to evaluate the therapeutic potential of FMT in treating IBD.^147-149^

### 10.3. Irritable bowel syndrome

The involvement of the microbiome in disease is well illustrated by its association with irritable bowel syndrome (IBS).^150^ ^,^ ^151^ IBS is defined by persistent, recurring abdominal pain associated with bowel movements or alterations in stool frequency or consistency, occurring in the absence of alarm symptoms.^152^ Multiple studies have reported distinct variations in the fecal microbiota of individuals with IBS compared to healthy controls.^153^ In particular, individuals with IBS often show reduced levels of Bifidobacterium and Lactobacillus, along with an elevated proportion of Firmicutes. Furthermore, some studies have reported alterations in the small intestinal microbiota of IBS patients, suggesting that small intestinal bacterial overgrowth may play a role in the disorder.^154^ ^,^ ^155^ While the majority of studies have focused on adults, Saulnier et al. discovered distinct microbiome profiles in children with IBS compared to healthy controls. Using a select group of key bacterial species, they successfully classified IBS subtypes with an impressive accuracy of 98.5%.^156^

Additional evidence supporting the gut microbiome’s involvement in IBS arises from the development of post-infectious IBS, characterized by the persistence of IBS-like symptoms following bacterial or viral gastroenteritis.^157^ Proposed pathophysiological mechanisms include enteroendocrine cell hyperplasia, elevated T-lymphocyte infiltration, and increased intestinal permeability subsequent to infection. ^158^ ^,^ ^159^

To elucidate the contribution of gut microbiota to IBS, multiple studies have examined the impact of probiotic supplementation. Findings from meta-analyzes and systematic reviews indicate that probiotics are generally superior to placebos in alleviating IBS symptoms.^160-162^ However, findings vary significantly due to differences in sample sizes, study designs, and probiotic strains, complicating comparisons.^72^ To delineate the specific probiotic strains beneficial for IBS symptom relief, Ortiz-Lucas et al. conducted a review of 10 randomized controlled trials. Their analysis revealed that formulations containing *Bifidobacterium breve*, *Bifidobacterium longum*, or *Lactobacillus acidophilus* were associated with significant reductions in pain scores, whereas combinations including *B.breve*, *B. longum* with *Lactobacillus casei*, or *Lactobacillus plantarum* demonstrated improvements in abdominal distension.^163^

Dietary changes also present a potential treatment avenue for IBS. Fermentable carbohydrates can be poorly absorbed and exacerbate symptoms. Consistent with this, a low FODMAP (fermentable oligosaccharides, disaccharides, monosaccharides, and polyols) diet has been shown to alleviate symptoms in adults with IBS.^164^ ^,^ ^165^ Chumpitazi et al.^166^ confirmed this response in pediatric IBS patients, noting specific microbial signatures linked to the diet's effectiveness. Notably, FODMAP responders had baseline microbiomes enriched with taxa capable of saccharolytic metabolism and related metabolic pathways.^166^

### 10.4. Malnutrition

Children suffering from malnutrition have been found to possess an underdeveloped intestinal microbiota compared to healthy peers, characterized by reduced alpha diversity and an abnormal overrepresentation of Proteobacteria. Like the bacterial microbiome, the gut virome also follows a dynamic and adaptable developmental course during early childhood. Disruptions in this normal maturation process particularly involving intestinal phages and eukaryotic viruses may contribute to an increased risk of developing severe acute malnutrition.^83^

### 10.5. Asthma and allergy

**Figure 4.**
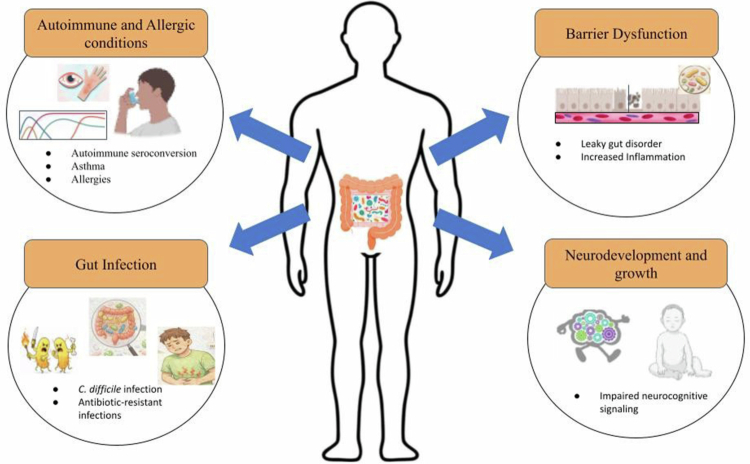
Imbalanced gut in the human microbiome. Created using https://BioRender.com.

Changes in the microbiome during infancy have been connected to the development of allergies and asthma. For example, newborns colonized with *Escherichia coli* are more likely to develop eczema, but infants colonized with *Clostridium difficile* are more likely to develop a variety of atopic diseases, such as allergic sensitization, recurrent wheezing, and eczema.^167^ Interestingly, colonization with *Clostridium difficile* at one month of age has been linked to an increased risk of developing asthma by 6 to 7 years of age.^168^ Moreover, antibiotic exposure during the first year of life has been linked to an increased risk of developing asthma, with the risk rising proportionally to the number of antibiotic courses administered. Experimental studies in mouse models have provided additional insights into these human findings. Germ-free mice with induced allergies displayed exacerbated symptoms compared to conventionally raised counterparts; notably, reintroduction of normal microbiota mitigated this severity, highlighting the critical role of the gut microbiota in modulating allergic responses (Figure 4).^169^ In a murine model of allergic airway inflammation (asthma), administration of *Lactobacillus reuteri* was shown to attenuate symptoms, whereas *Lactobacillus salivarius* had no such effect, emphasizing the strain-specific nature of probiotic action.^170^ Supporting these findings, Russell et al. found that exposure to vancomycin, unlike streptomycin, worsened the severity of allergic asthma in mice (Figure 4).^171^

### 10.6. Obesity

With rising prevalence rates, obesity has emerged as a major global health concern.^172^ Emerging evidence indicates that early-life microbial community development may influence the risk of childhood obesity. A recent study involving more than 900 infants revealed that delivery mode and gut microbiota composition particularly members of the *Lachnospiraceae* family modulated the relationship between maternal pre-pregnancy overweight status and the child’s body weight at 1 and 3 years of age.^173^ Another study linked low levels of *Bifidobacterium spp*. and high Staphylococcus aureus in infancy to a greater chance of being overweight by age seven.

Additional evidence for the microbiome’s involvement in obesity was provided by Cho et al., who demonstrated that administering low-dose antibiotics to young mice resulted in increased adiposity and disrupted metabolic pathways.^174^ Furthermore, Cox et al. demonstrated that administering low-dose penicillin at birth produced long-term alterations in body composition and amplified obesity induced by high-fat diets. Remarkably, transferring the microbiota from penicillin-treated mice to germ-free recipients reproduced these effects, underscoring the microbiome’s pivotal role in obesity development.^36^ ^,^ ^175^

## 11. Microbiome-targeted interventions and future directions for modulating pediatric health trajectories

Advances in microbiome-targeted therapies offer opportunities to modulate interacting microbial, immune, and metabolic pathways during critical developmental windows to improve pediatric health outcomes. Given the critical role of the microbiome in pediatric health, various therapeutic interventions are being explored to modulate the gut microbiome in order to improve health outcomes.

### 11.1. Probiotics and prebiotics

Probiotics and prebiotics have been extensively studied for their role in restoring microbial balance in children. Probiotics, particularly Bifidobacterium and Lactobacillus strains, support gut health and enhance immune function.^176^ Beyond gut benefits, probiotic supplementation has also been linked to improved cognitive outcomes in conditions such as HIV, Parkinson’s disease, fibromyalgia, major depressive disorder, Alzheimer’s disease, and mild cognitive impairment.^177^ Prebiotics, including dietary fibers and human milk oligosaccharides (HMOs), promote the growth of beneficial bacteria and increase gut microbial diversity.^176^ Compounds such as inulin and fructooligosaccharides stimulate the proliferation of microbes like *Faecalibacterium prausnitzii* and *Akkermansia*, while pectin supplementation in colorectal cancer models has been shown to enrich butyrate-producing families such as Lachnospiraceae and Ruminococcaceae.^178^ In-utero ingestion of vernix caseosa supports healthy gut microbiome development by promoting colonization of branched-chain fatty acid utilizing commensal bacteria such as *Bifidobacterium*, *Bacteroides*, and *Propionibacterium* species.

### 11.2. Fecal microbiota transplantation (FMT)

Fecal microbiota transplantation (FMT) is an emerging therapeutic strategy designed to restore gut microbial diversity and function by transferring beneficial microbiota from healthy donors to affected individuals.^179^ It is well established for the treatment of recurrent *C. difficile* infection and is being investigated for a wide range of conditions linked to gut imbalanced gut, including ulcerative colitis, irritable bowel syndrome, sepsis, metabolic disorders, neurodevelopmental conditions such as autism, and neurodegenerative diseases such as Parkinson’s disease and Alzheimer’s disease. Current studies suggest that FMT is generally safe in the short term, with most adverse effects limited to mild, self-limiting GI symptoms such as abdominal pain, bloating, diarrhea, constipation, nausea, or vomiting. Its use in pediatric populations remains limited, though early studies have explored applications in IBD and autism, with long-term safety and efficacy still under evaluation.^180^ ^,^ ^181^ Additionally, FMT has shown benefits beyond the gut, such as improving cognitive function in patients with hepatic encephalopathy, reinforcing the role of a balanced gut microbiota (eubiosis) in overall GI, immune, and neurological health.^177^

### 11.3. Dietary interventions

Diet and lifestyle are key determinants of gut microbiota composition and function. Dietary diversity shapes microbial fermentation and the production of metabolites that influence host health. High-fat diets have been associated with microbiota alterations that may promote GI cancers, whereas high-salt diets appear to enhance anti-tumor immunity by suppressing myeloid-derived suppressor cell differentiation. Such diets also increase *Bifidobacterium bifidum* abundance and raise levels of the microbial metabolite mercuric acid, which strengthens NK cell cytotoxicity and improves the efficacy of anti-PD-1 immunotherapy, particularly under conditions of elevated interferon-gamma.^178^ Dietary modifications, particularly increasing fiber intake and promoting a plant-based diet, have been shown to positively influence the gut microbiome. Studies suggest that a diet rich in fruits, vegetables, and whole grains may enhance microbial diversity and reduce the risk of diseases such as obesity, allergies, and inflammatory bowel disease in children.

## 12. Conclusions

In this review, we summarize the current insights into the development of the pediatric microbiome, its influence on brain development and function via the gut-brain axis, and how imbalanced gut can contribute to disease progression. Imbalanced gut in early life has been linked to a range of pediatric diseases, including obesity, allergies, inflammatory bowel disease, and neurodevelopmental disorders. Understanding the factors that shape the microbiome during early childhood and exploring strategies to modulate it may provide valuable insights into the prevention and treatment of these conditions. The gut microbiome is closely linked to GI, metabolic, immune, and neurodevelopmental disorders, with early-life disruptions having lasting effects on child health. In pediatrics, this knowledge carries important clinical value: microbial patterns may serve as diagnostic or prognostic biomarkers, while interventions such as probiotics, prebiotics, dietary changes, or FMT hold therapeutic promise. Incorporating microbiome insights could help pediatricians move toward more personalized care, enabling earlier intervention and improved long-term outcomes in at-risk children. Future research into targeted microbiome-based therapies, such as probiotics, prebiotics, and FMT, holds promise for improving pediatric health.
